# The Week's Parliament and Publications

**Published:** 1910-07-16

**Authors:** 


### The Week's Parliament and Publications.

### THE EMPLOYMENT OF CHILDREN.

In July 1909 a Departmental Committee was appointed to inquire into the operation of the Employment of Children Act, 1903, and to consider whether any and what further legislation, regulation, or restriction is required in respect of street trading and other employments dealt with in the Act. The committee are divided in their opinions, and a Majority and Minority Report have been published. The former is signed by seven members, and recommends that street trading by boys under 17 and girls under 18 be prohibited. The Minority Report, which is signed by four members, recommends that powers be given to local authorities to prohibit street trading up to the age of 18, *where it can be shown that other suitable forms of employment are available*; in cases where the local authorities grant licences to boys under 18 and over 14 years this report recommends that it be made a condition of the licence that the boys should attend technical and continuation classes.

### PUBLIC HEALTH IN SCOTLAND.

A number of matters of medical interest are dealt with in the fifteenth annual report of the Local Government Board for Scotland which relates to the year 1909. In the section concerning the administration of the Poor Law the report states that on several occasions the Board have been asked whether the medical officer of a parish is bound, in respect of his official salary, to afford medical assistance to persons in receipt of old-age pensions. The Board replied that, when a person in receipt of an old-age pension applies for medical relief, and, in the opinion of the inspector of poor or parish council, is unable to pay for the same, it is the duty of the inspector of poor to give the applicant an order entitling him to the services of the medical officer. Such services are covered by the salary which the medical officer receives under the Poor Law.

With regard to the Notification of Births Act, 1907, the Board communicated, at the end of last year, with all the more important burghs and with the principal districts that had not adopted the Act urging them to consider the advisability of availing themselves of the power given by the Act for the early notification of births. Most of the local authorities still have the matter under consideration, but in some cases replies have been received stating that the local authority do not consider it necessary to adopt the Act, which view appears also to be held by the medical officers of health.

The number of samples examined during the year under the Sale of Food and Drugs Acts was 7,204, of which 10.5 per cent. were not up to standard. Milk was the article most frequently adulterated, the percentage of samples not up to standard being 14.2. The drugs examined included the following: Cream of tartar, 108 samples examined, 4 adulterated; tartaric acid, 43 examined, 16 adulterated, 14 of which were reported to be "of doubtful purity"; camphorated oil, 20 samples examined, 1 of which was reported to be "of doubtful purity"; and 1 sample of mustard oil, which was found to be adulterated. Prosecutions were instituted in respect of 411 samples, and convictions were obtained in 333 cases, the fines and expenses recovered amounting to £1,133 11s. 3d. Of the informal or "test" samples taken, 17.1 per cent. were found not to be up to standard, which seems to be an unusually high percentage.

Regarding vaccination, there has been an increase in the number of defaulters as compared with previous years. This appears to be attributable to several causes, one of which is a general impression among the people that vaccination is no longer compulsory, which impression is partly due to a misunderstanding of the terms of the Vaccination (Scotland) Act, 1907. The total number of tubes of lymph supplied in 1909 to parochial vaccinators and poorhouse authorities was 2,677, which consisted entirely of glycerinated calf-lymph.

Some interesting information is contained in the report concerning the value of wood and iron hospitals. Eleven country officers and the officer of an asylum state their experience of buildings of this kind. The view of the county medical officer for Stirling and Dumbarton is that buildings of wood and iron are particularly suitable if for any reason, such as their reservation for smallpox, they have to stand closed for lengthened periods of time. His opinion is that a brick or stone building with plastered walls is much more under the influence of damp when standing empty and without fires. A wood and iron building is very easily prepared for immediate occupation, but in winter the wards are difficult to keep warm, and in summer they are apt to be too warm. They require much better window ventilation than is sometimes provided.—*Fifteenth Annual Report of the Local Government Board for Scotland* (Cd. 5228). Price 1s. 8d.

